# Nitric oxide acts as an antioxidant and inhibits programmed cell death induced by aluminum in the root tips of peanut (*Arachis hypogaea* L.)

**DOI:** 10.1038/s41598-019-46036-8

**Published:** 2019-07-02

**Authors:** Huyi He, Thet Lwin Oo, Wenjing Huang, Long-Fei He, Minghua Gu

**Affiliations:** 1 College of Agronomy, Guangxi University, Nanning, 530004 P.R. China; 2 Cash Crops Research Institute, Guangxi Academy of Agricultural Sciences, Nanning, 530007 P.R. China; 3 Guangxi Key Laboratory for Agro-Environment and Agro-Product Safety, Nanning, 530004 P.R. China

**Keywords:** Plant physiology, Abiotic

## Abstract

Aluminum (Al) causes programmed cell death (PCD) in plants. Our previous studies have confirmed that nitric oxide (NO) inhibits Al-induced PCD in the root tips of peanut. However, the mechanism by which NO inhibits Al-induced PCD is unclear. Here the effects of NO on mitochondrial reactive oxygen species (ROS), malondialdehyde (MDA), activities of superoxide dismutase (SOD) and ascorbate peroxidase (APX), expression of alternative oxidase (*AhAOX*) and cytochrome oxidase (*AhCOX*) were investigated in peanut (*Arachis hypogaea* L.) root tips treated with Al. The results showed that Al stress induced rapid accumulation of H_2_O_2_ and MDA and increased the ratio of SOD/APX. The up-regulation of *AhAOX* and *AhCOX* expressions was not enough to inhibit PCD occurrence. Sodium nitroprusside (SNP, a NO donor) decreased the ratio of SOD/APX and eliminated excess H_2_O_2_ and MDA, thereby inhibiting Al-induced PCD in the root tips of peanut. The expression of *AhAOX* and *AhCOX* was significantly enhanced in Al-induced PCD treated with SNP. But cPTIO (a NO specific scavenger) supply had the opposite effect. Taken together, these results suggested that lipid peroxidation induced by higher levels of H_2_O_2_ was an important cause of Al-induced PCD. NO-mediated inhibition of Al-induced PCD was related to a significant elimination of H_2_O_2_ accumulation by decreasing the ratio of SOD/APX and up-regulating the expression of *AhAOX* and *AhCOX*.

## Introduction

As a major factor, aluminum (Al) toxicity limits crop productivity in acid soil. Al inhibits root elongation growth and disrupts the uptake of nutrient and water in plants. Al enhanced the production of reactive oxygen species (ROS), leading to mitochondrial respiration inhibition and ATP depletion in plant cells^1^. Al exclusion and tolerance mechanisms are associated with mitochondrial metabolism, especially organic acid transport and mitochondrial activity^2^. Programmed cell death (PCD) is a process of cellular suicide controlled by genes. Al toxicity may be the result of Al-induced PCD in the root tips of barley^3^. The negative regulation of PCD alleviated Al toxicity in yeast^4^. Our previous researches showed that Al induced mitochondria-dependent PCD in Al-sensitive peanut cultivar rapidly^5^.

Nitric oxide (NO) has been considered as a signal regulator involved in plant growth and stress tolerance. Al stress changes the homeostasis of endogenous NO in plants^6^. Exogenous NO donor sodium nitroprusside (SNP) treatment can alleviate Al toxicity by ameliorating effectively Al-induced mitochondrial respiratory dysfunction in wheat root tips^7^. The former studies of our group had showed that NO suppresses PCD induced by Al in peanut root tips^8^. NO may be an antioxidant to postpone PCD in barley aleurone layers^9^. However, the mechanism by which NO inhibits Al-induced PCD is unclear.

As an important power station, mitochondrion is mainly responsible for electron transport, oxidative phosphorylation, and energy metabolism in animal and plant cells. Higher concentrations of Al treatment opened the mitochondrial membrane permeability transition pore (MPTP) and released cytochrome c (Cyt c) into the cytoplasm, thereby induced PCD in peanut root tips^10^. In *Arabidopsis thaliana*, Al toxicity induced mitochondria-dependent PCD^11^. Oxidative stress increased ROS generation, the opening of MPTP, and activation of proteases, leading to the occurrence of PCD in *Arabidopsis* cells^12^. In the early stages of PCD in heat-shocked tobacco BY-2 cells, cytosolic ascorbate peroxidase (cAPX) was damaged and ROS were generated with the injury of mitochondrial metabolism^13^. ROS can trigger Cyt c release and the activation of caspase-like proteases is required to cause cell death^14^. As the regulatory center of PCD, the structure and function of mitochondria also played a vital role during Al-induced PCD in peanut^15^. In the root tip cells of peanut, Al-induced ROS burst activated mitochondria-dependent PCD^16^. The over-reduction of mitochondrial electron transport chain is the primary sources of ROS production in plant root tip cells^17^. Alternative oxidase (AOX) is the terminal oxidase of the cyanide-resistant respiration pathway in the mitochondrial respiratory chain of plants. Cytochrome c oxidase (COX) is a kind of cytochrome oxidase located in the terminal of the mitochondrial respiratory chain. Under stress condition, AOX and COX can change the level of respiratory metabolism, scavenge ROS, and regulate cell function. The suppression of NO on Al-induced PCD was associated with the improvement of mitochondrial physiological properties in peanut root tips^18^. Whether mitochondrial ROS are involved in the inhibition of Al-induced PCD by NO has not been reported.

In the present study, to explore the mitochondrial pathway of NO regulating Al-induced PCD, the effects of treatment with NO donor and NO specific scavenger on mitochondrial ROS, malondialdehyde (MDA), activities of superoxide dismutase (SOD) and ascorbate peroxidase (APX), expression of *AhAOX* and *AhCOX* under Al-induced PCD in the root tips of peanut were investigated. These results indicated that excess H_2_O_2_-induced lipid peroxidation was an important cause of Al-induced PCD. NO-mediated inhibition of PCD induced by Al was related to a significant decrease in the ratio of SOD/APX and the up-regulation of *AhAOX* and *AhCOX* expressions.

## Results

### Evaluation of mitochondrial function

To evaluate the mitochondrial function, the membrane potential and Cyt c value were detected. The mitochondrial membrane potential can be expressed as the fluorescence intensity of the fluorescent probe Rh-123. The results showed that the fluorescence intensity of the control and Al treatment were 0.89 and 0.56, respectively. After Al treatment, Cyt c value decreased from 21.4 nmol·μg^−1^ Pro to 9.2 nmol·μg^−1^ Pro. This indicated that the mitochondrial function was pure, integral, and viable.

### Effects of Al on mitochondrial O_2_^.−^, H_2_O_2_, and lipid peroxidant

**Figure 1:**
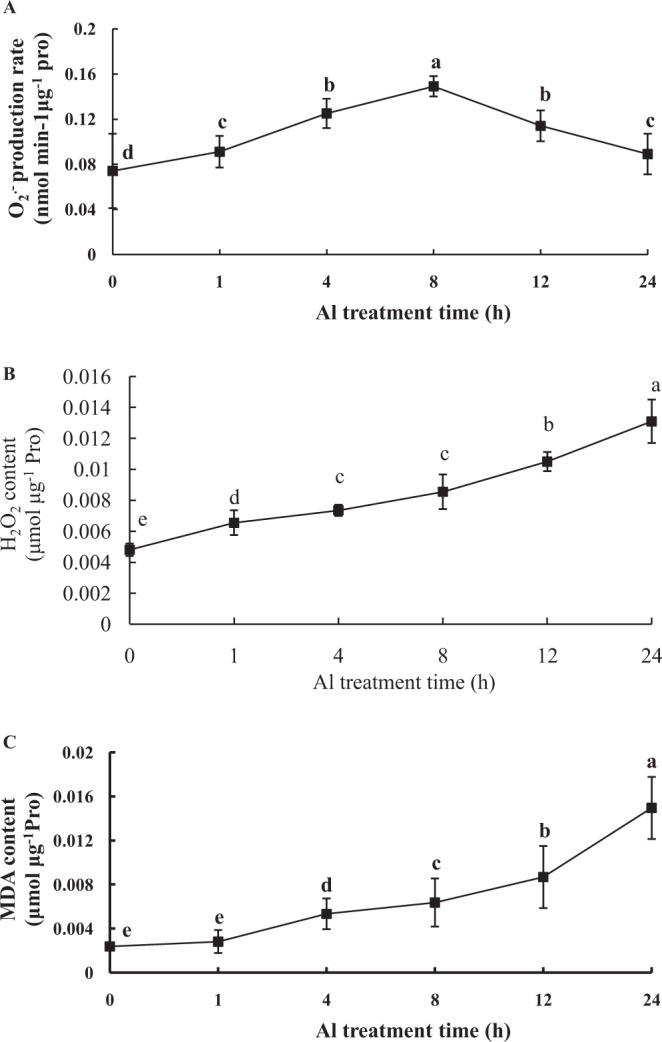
Changes of mitochondrial O_2_^.−^ production rate (**A**), H_2_O_2_ content (**B**) and MDA content (**C**) in peanut roots under 100 μmol·L^−1^ AlCl_3_ treatment for 0 h (control), 1 h, 4 h, 8 h, 12 h, and 24 h. Each data in figure represents the mean ± SD of three experiments. Different lower case letters indicate significance at *P* < 0.05 compared to the control.

When plants are exposed to adversity stress, a lot of ROS are produced and excess ROS do harm to plants. To study the effects of Al on mitochondrial ROS and lipid peroxidant, peanut seedlings were exposed to 100 μmol·L^−1^ AlCl_3_ solution at different times. As Al treatment time extended, O_2_^.−^ production rate increased sharply to the peak at 8 h and then decreased slowly (Fig. 1A). Compared to the control, the mitochondrial O_2_^.−^ production rate reached a peak in the root tips of peanut at 8 h of Al treatment. As shown in Fig. 1B, the mitochondrial H_2_O_2_ content of peanut root tips was increased after Al treatment. Al treatment for 1 h increased significantly H_2_O_2_ content and then kept up at a higher level compared with the control. MDA content is always used as an indicator to estimate the level of lipid peroxidation. The results showed that the MDA content was increased sharply as Al treatment time increased (Fig. 1C). Compared with the control, MDA content of mitochondria was increased by 2.2 fold at the time of 4 h Al treatment in the root tips of peanut. At 12 h and 24 h, MDA content increased by 3.6 fold and 6.3 fold, respectively. MDA content was increased significantly in a time-dependent manner.

### Effects of Al on mitochondrial antioxidase activities

**Figure 2:**
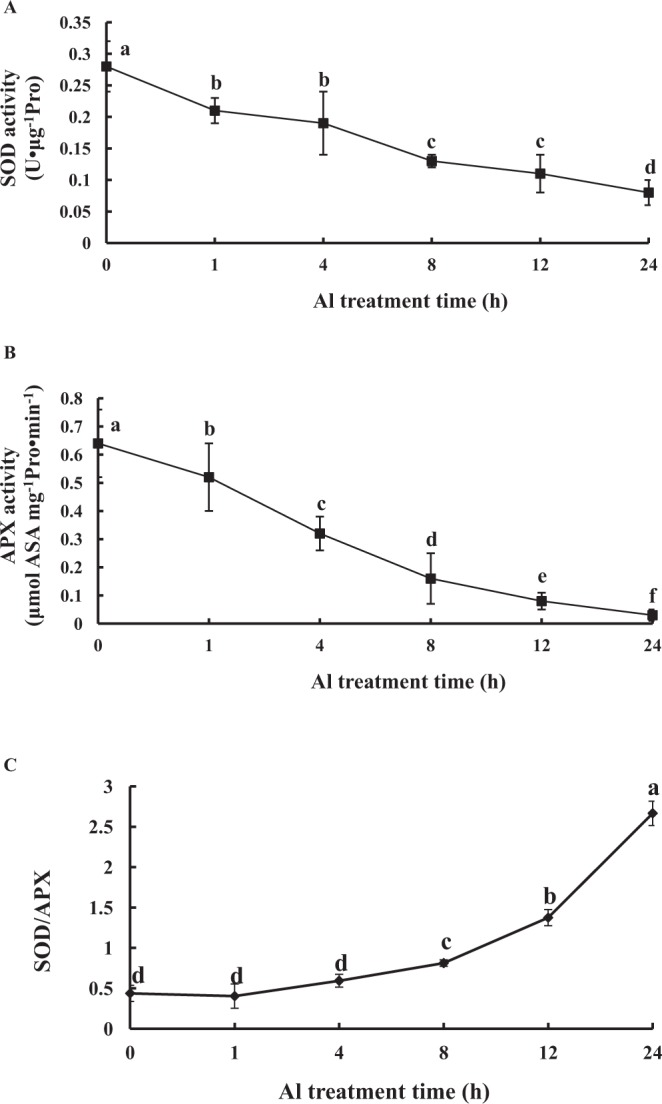
Changes of mitochondrial SOD activity (**A**), APX activity (**B**), and SOD/APX (**C**) from peanut roots under 100 μmol·L^−1^ AlCl_3_ treatment for 0 h (control), 1 h, 4 h, 8 h, 12 h, and 24 h. Each data in figure represents the mean ± SD of three experiments. Different lower case letters indicate significance at *P* < 0.05 compared to the control.

To study the effects of Al on mitochondrial antioxidase activities, the activities of SOD and APX were measured. The activity of SOD was gradually decreased as Al treatment time prolonged (Fig. 2A). Compared with the control, SOD activity of peanut root tips was decreased by 32.1% at 4 h Al treatment. At 12 h and 24 h, SOD activity had dropped by 60.7% and 71.4%, respectively. The activity of APX was sharply decreased as Al treatment time increased (Fig. 2B). Compared to the control, the activity of APX in the root tips of peanut was reduced by 50% at 4 h of Al treatment. At 12 h and 24 h, APX activity had dropped by 87.5% and 95.3%, respectively. As Al treatment time increased, the ratio of SOD/APX was rapidly increased (Fig. 2C).

### Relationship between root cell death and H_2_O_2_, MDA, SOD/APX in peanut root tips

**Figure 3:**
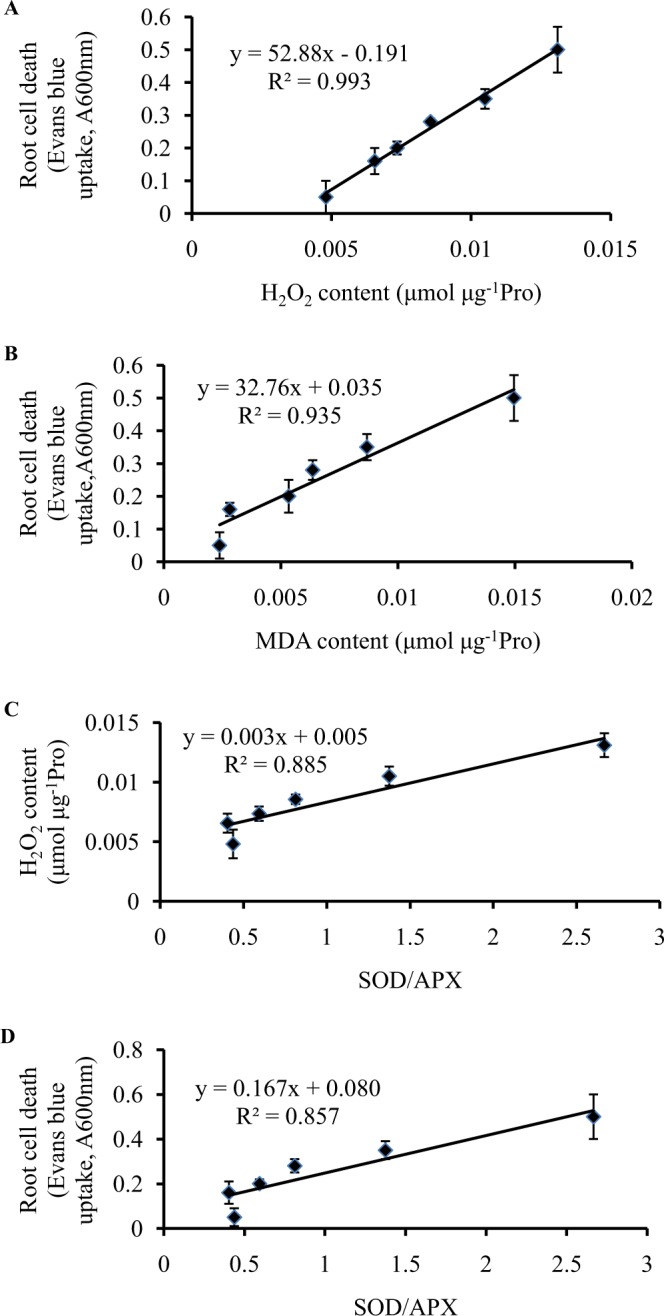
Relationship between root cell death and H_2_O_2_ content (**A**), MDA content (**B**), respectively. Relationship between SOD/APX and H_2_O_2_ content (**C**), cell death (**D**), respectively in peanut root tips.

As shown in Fig. 3A,C, H_2_O_2_ content in mitochondria of the peanut root tips was significantly positively correlated with not only cell death (R^2^ = 0.993) but also SOD/APX (R^2^ = 0.885). However, the correlation between cell death and superoxide was very poor (R^2^ = 0.044). There was a significantly positive relationship between lipid peroxidation and cell death in peanut root tips (R^2^ = 0.935) (Fig. 3B). Fig. 3D showed that SOD/APX was significantly positively correlated with cell death in the root tips of peanut (R^2^ = 0.857).

### Effects of NO on O_2_^.−^, H_2_O_2_, and MDA in the root tips of peanut

**Figure 4:**
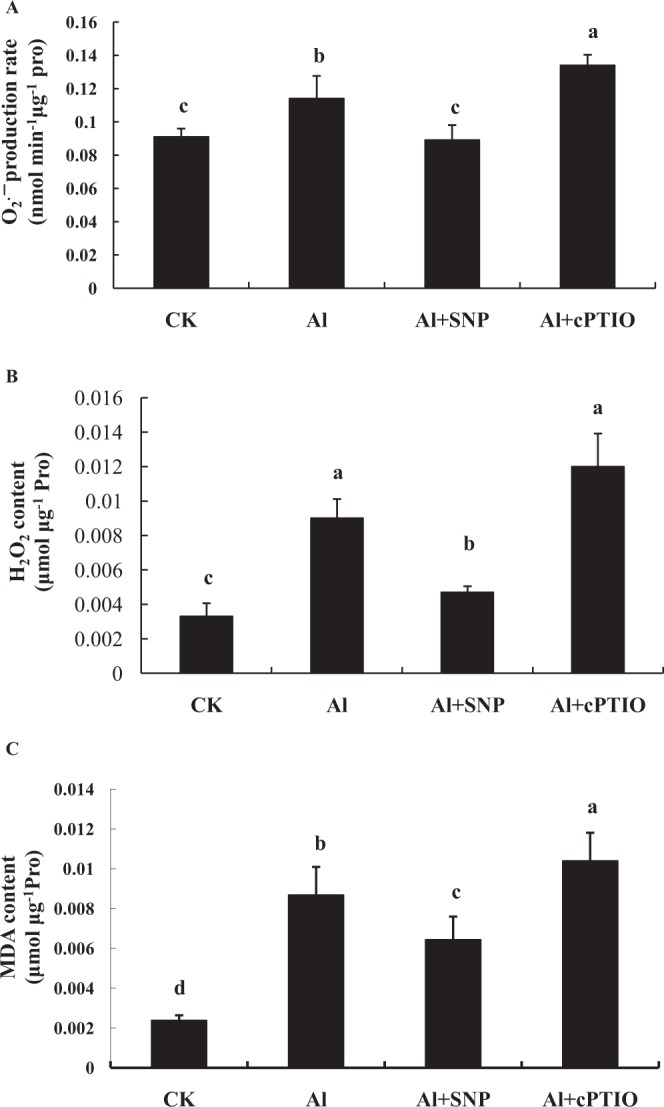
Changes of mitochondrial O_2_^.−^ production rate (**A**), H_2_O_2_ content (**B**), and MDA content (**C**) from peanut roots treated with 0.1 mmol·L^−1^ CaCl_2_ (pH 4.2) (CK), 100 μmol·L^−1^ AlCl_3_ (Al), 100 μmol·L^−1^ AlCl_3_ + 200 μmol·L^−1^ SNP (Al + SNP), and 100 μmol·L^−1^ AlCl_3_ + 50 μmol·L^−1^ cPTIO (Al + cPTIO) for 12 h. Each data in figure represents the mean ± SD of three experiments. Different lower case letters indicate significance at *P* < 0.05 compared to the control.

To study the effects of NO on mitochondrial ROS and lipid peroxidation, peanut seedlings were exposed to different chemical treatments. As shown in Fig. 4A, NO decreased mitochondrial O_2_^.−^ production rates in the root tips of peanut. Compared with Al treatment alone, SNP effectively inhibited the production of mitochondrial O_2_^.−^, while cPTIO (a NO specific scavenger) significantly increased the production rate of O_2_^.−^. As shown in Fig. 4B, NO inhibited H_2_O_2_ production by mitochondria in the root tips of peanut. Compared with Al treatment, SNP significantly inhibited the production of mitochondrial H_2_O_2_, while cPTIO intensified H_2_O_2_ content. As shown in Fig. 4C, NO decreased mitochondrial MDA content in peanut root tips. Compared to Al treatment alone, SNP significantly reduced mitochondrial MDA content in the root tips of peanut, while cPTIO significantly increased MDA content.

### Effects of NO on SOD, APX, and SOD/APX in the root tips of peanut

**Figure 5:**
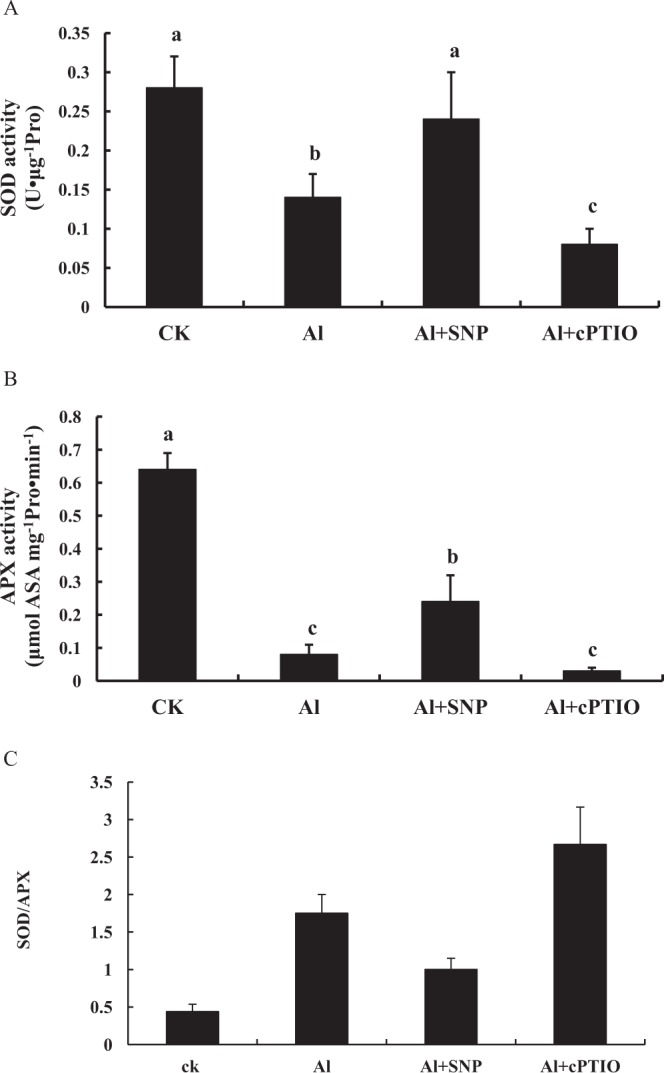
Changes of mitochondrial SOD activity (**A**), APX activity (**B**), and SOD/APX (**C**) from peanut roots treated with 0.1 mmol·L^−1^ CaCl_2_ (pH 4.2) (CK), 100 μmol·L^−1^ AlCl_3_ (Al), 100 μmol·L^−1^ AlCl_3_ + 200 μmol·L^−1^ SNP (Al + SNP), and 100 μmol·L^−1^ AlCl_3_ + 50 μmol·L^−1^ cPTIO (Al + cPTIO) for 12 h. Each data in figure represents the mean ± SD of three experiments. Different lower case letters indicate significance at *P* < 0.05 compared to the control.

To study the effects of NO on mitochondrial antioxidase activities, the activities of SOD and APX were measured under different chemical treatments. As shown in Fig. 5A, Al treatment inhibited the activity of mitochondrial SOD in the root tips of peanut. Compared to Al treatment alone, SNP significantly increased the activity of SOD, while cPTIO significantly reduced the activity of SOD. As shown in Fig. 5B, Al treatment inhibited the activity of mitochondrial APX in the root tips of peanut. Compared to Al treatment alone, SNP significantly increased the activity of APX, while cPTIO significantly reduced the activity of APX. The addition of SNP decreased the ratio of SOD/APX, which was increased by Al treatment, while cPTIO supplement increased the ratio of SOD/APX (Fig. 5C).

### Effects of NO on *AhAOX* and *AhCOX* expression during Al-induced PCD

**Figure 6:**
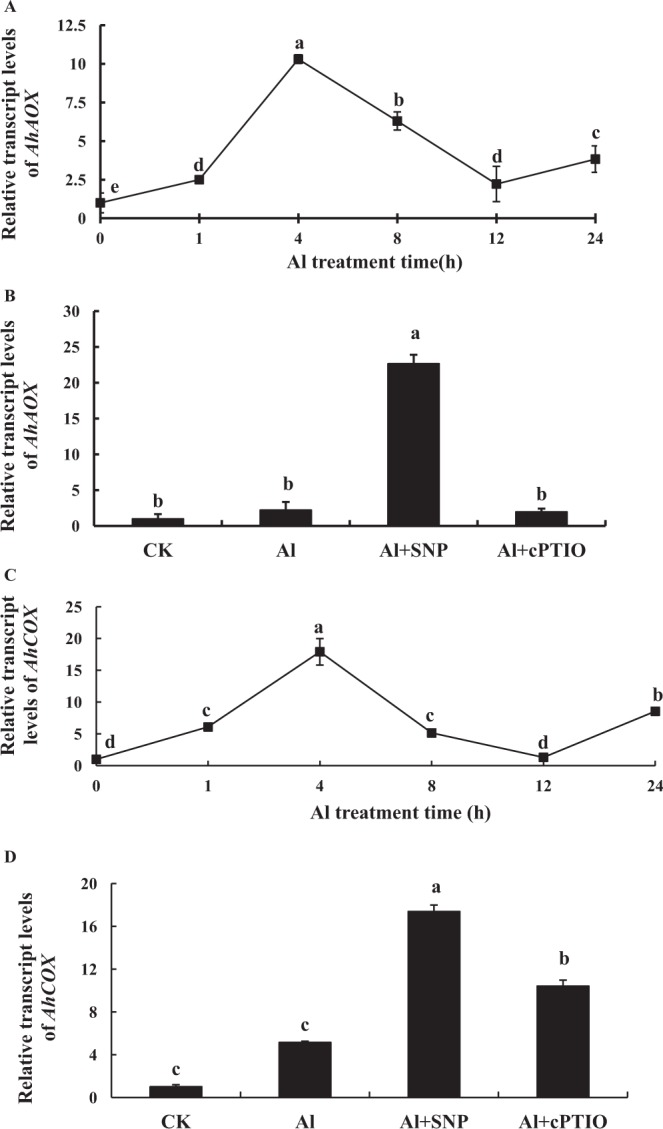
The expression of alternative oxidase (*AhAOX*) and cytochrome oxidase (*AhCOX*) at different times (**A**,**C**) and different treatments for 12 h (**B**,**D**) under Al stress, respectively. Each data in figure represents the mean ± SD of three experiments. Different lower case letters indicate significance at *P* < 0.05 compared to the control.

The expression of *AhAOX* was rapidly increased and then slowly decreased under Al stress (Fig. 6A). After 1 h of Al treatment, the expression of *AhAOX* was increased, indicating that Al boosted the expression of this gene. At 4 h Al treatment, the expression of this gene was the highest, which was 10.3 times than that of the control. The expression of *AhAOX* was then reduced. The expression of this gene at 12 h Al treatment was 2.22 times of the control. As shown in Fig. 6B, compared to Al treatment alone, the addition of SNP increased significantly the expression of *AhAOX*, an increase of 919.65%; cPTIO supplement decreased the expression of *AhAOX*, but there was no significant difference compared with Al treatment alone.

The expression of *AhCOX* was rapidly increased and then slowly decreased under Al treatment (Fig. 6C). After 1 h of Al treatment, *AhCOX* expression was increased, indicating that Al motivated the expression of this gene. At 4 h Al treatment, the expression of this gene was the highest, which was 17.92 times than that of the control. Then the expression of *AhCOX* was gradually decreased. The expression of this gene at 12 h was 1.30 times than that of the control. As shown in Fig. 6D, compared with Al treatment alone, the addition of SNP significantly increased the expression of *AhCOX*, an increase of 237.92%, while the expression of *AhCOX* for cPTIO supplement was significantly higher than that of Al treatment.

### Hierarchical cluster analysis of ROS in Al-induced PCD in the root tips of peanut

**Figure 7:**
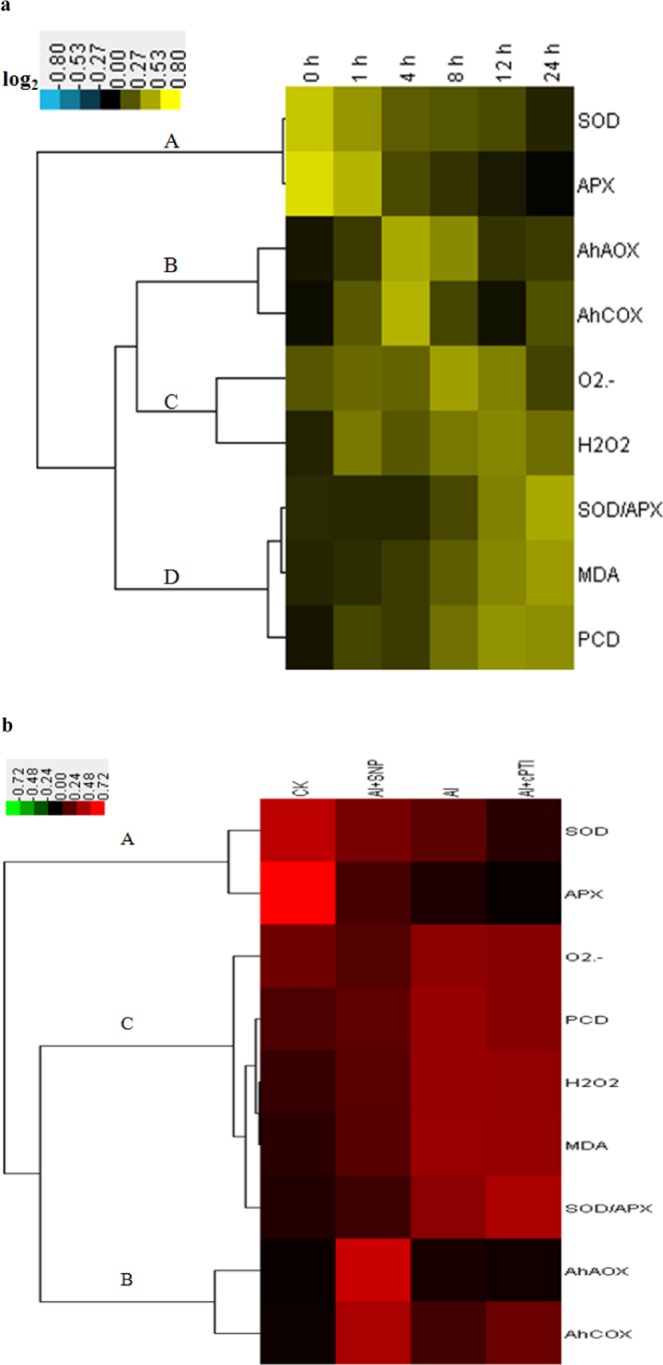
(**a**) Hierarchical cluster analysis of mitochondrial physiological parameters (SOD and APX (A), *AhAOX* and *AhCOX* (B), O_2_^.−^ and H_2_O_2_ (C), SOD/APX, MDA, and cell death (D)) in peanut root tips under different Al treatment time. (**b**) Hierarchical cluster analysis of mitochondrial physiological parameters (SOD and APX (A), *AhAOX* and *AhCOX* (B), O_2_^.−^, H_2_O_2_, SOD/APX, MDA, and cell death (C)) in the root tips of peanut under different treatments. Resulting tree diagram was obtained by using Cluster 3.0 software and Java Treeview. The cluster color bar was shown as log_2_ fold change.

Based on the data of mitochondrial O_2_^.−^, H_2_O_2_, MDA (Fig. 1), SOD, APX, SOD/APX (Fig. 2), *AhAOX* (Fig. 6A), *AhCOX* (Fig. 6C), and cell death in the root tips of peanut with different Al treatment time, hierarchical cluster was conducted to analyze the interaction between NO and ROS on peanut response to Al stress. The results indicated that Al stress inhibited the activities of SOD and APX (Fig. 7a-A), increased the ratio of SOD and APX, up-regulated the expression of *AhAOX* and *AhCOX* (Fig. 7a-B), promoted the accumulation of H_2_O_2_, O_2_^.−^ (Fig. 7a-C), and MDA, resulting in Al-induced PCD in peanut root tips (Fig. 7a-D). Under Al stress, the physiological parameters are clustered well to four groups (A–D). There is a causal relationship between group A and D. Group B and C are paralleled.

### Hierarchical cluster analysis of ROS in NO inhibiting Al-induced PCD in the root tips of peanut

Based on the data of mitochondrial O_2_^.−^, H_2_O_2_, MDA (Fig. 4), SOD, APX, SOD/APX (Fig. 5), *AhAOX* (Fig. 6B), *AhCOX* (Fig. 6D), and cell death in the root tips of peanut with different treatments, hierarchical cluster was used to analyze the interaction between NO and ROS on peanut response to Al stress. The results indicated that NO donor SNP promoted the activities of SOD and APX (Fig. 7b-A), decreased the ratio of SOD and APX, up-regulated the expression of *AhAOX* and *AhCOX* (Fig. 7b-B), reduced the accumulation of H_2_O_2_ and O_2_^.−^ (Fig. 7b-C), leading to inhibition of Al-induced PCD in the root tips of peanut. cPTIO supply had the opposite effects. During NO inhibiting Al-induced PCD, the physiological parameters are clustered well to three groups (A–C). There is causal relationship between group A and C. Group B and C are paralleled. Group C is also divided into two subgroups, which have a more direct causal relationship.

## Discussion

Excessive H_2_O_2_ -induced lipid peroxidation is an important cause of Al-induced PCD. H_2_O_2_ is an unstable and strong oxidant. When Fe^2+^ is present, the Fenton reaction (H_2_O_2_ + Fe^2+^ → OH^−^ + OH^•^ + Fe^3+^) occurs. As one of the most active ROS, the formative hydroxyl radical (OH^•^) is highly harmful to all biomolecules in plants including DNA, RNA, lipids, and proteins. So the excess H_2_O_2_ must be eliminated promptly. ROS closely related with Al-induced PCD in the root tips of peanut is H_2_O_2_ rather than O_2_^.−^ (Fig. 3A). Cadmium (Cd) induced the accumulation of H_2_O_2_ in the roots of *Pinus sylvestris* L., induced xylem formation and accelerated senescence^19^. Low concentrations of Al stimulated PLC and PLD signaling pathways to lead to ROS production, followed by the caspase-like protease to execute cell death^20^. Moreover, the results of correlation analysis indicated that lipid peroxidation induced by higher levels of H_2_O_2_ might be an important cause of Al-induced PCD (Fig. 3A,B). NO can combine with O_2_^.−^ to form peroxynitrite (ONOO^−^), which can induce cell death. Because O_2_^.−^ was not major ROS during Al-induced PCD, in fact, ONOO^−^ was rarely generated.

### Nitric oxide mediates inhibition of Al-induced PCD by decreasing the ratio of SOD/APX to scavenge excess H_2_O_2_

The decrease of SOD/APX ratio contributed to the elimination of H_2_O_2_ (Fig. 3C), so the increase of SOD/APX ratio may be associated with Al-induced PCD in peanut roots (Fig. 3D). NO donor SNP enhances the antioxidant capacity of wheat seedlings under Al stress^21^. NR-dependent NO production alleviated Al-induced oxidative stress in the roots of red bean^22^. NO suppressed *Cassia tora* root sensitivity to Al by inactivating the cell wall peroxidase activity and reducing H_2_O_2_ production^23^. As an antioxidant, NO increased the activities of SOD and CAT, delay PCD in barley aleurone layers^9^. In the induction of plant cell death, there is crosstalk and synergistic action between NO and H_2_O_2_^24^. NO treatment reversed Al-induced reactive oxygen species toxicities by promoting the expression of antioxidant enzymes^25^. The decrease of NO level promoted the accumulation of ROS and induced the expression of pathogen-related proteins (PRs) to protect cells from Cd toxicity^26^. NO and ROS can induce cell death alone or synergistically^27^. In the present study, Al stress decreased SOD and APX activity and raised membrane lipid peroxidation in peanut apex, while NO activated antioxidant enzymes (SOD, APX) system to protect the peanut root tip from ROS damage. The result is consistent with the findings of Wang and Yang^28^. Because the decreasing range of APX activity was larger than that of SOD activity, excessive H_2_O_2_ could not be removed in time. With the prolonging of Al treatment time, Al stress increased H_2_O_2_ production and MDA accumulation, which was related to the rise of SOD/APX ratio. The linear relationship between SOD/APX and H_2_O_2_ content also clearly indicated that the content of H_2_O_2_ increased with the rise of SOD/APX ratio (Fig. 3C). Acute stress generated ROS and reactive nitrogen species (RNS) and lead to APX degradation. The regulation of APX mediated by NO may be a redox sensor of oxidative stress^29^. H_2_O_2_ alleviated salt-induced oxidative stress by modulating APX and SOD activities in cotton^30^. Similar to the result of Fan *et al*.^31^, NO up-regulated the activities of SOD and APX. But the rising range of APX activity was larger than that of SOD activity, resulting in the decrease of SOD/APX ratio. By decreasing the ratio of SOD/APX, SNP eliminated excess H_2_O_2_ and decreased MDA, thereby inhibiting Al-induced PCD in the root tips of peanut. But cPTIO supply had the opposite effect.

### Inhibition of Al-induced PCD by NO is related to the enhancement of *AhAOX* and *AhCOX* expressions

AOX can be adapted to the environmental changes by regulating its own structure, which plays an important part in plant physiology^32^. AOX can effectively reduce the production of mitochondrial ROS in plant cells and decrease cell injury^33^. The lack of mitochondrial AOX increased susceptibility to PCD in transgenic plants, but induction of mitochondrial AOX prevented PCD by down-regulating the cytochrome pathway^34,35^. Salicylic acid and H_2_O_2_ treatment up-regulated the expression of AOX in wild type tobacco, reduced ROS accumulation in mitochondria and delayed PCD occurrence^36^. AOX acts as a buffer that determines the threshold of PCD induction^37,38^. The expression of AOX was significantly up-regulated in tobacco suspension cells treated with 500 μmol·L^−1^ AlCl_3_. Overexpression of AOX could enhance the tolerance of tobacco suspension cells to Al stress^11^. The PCD degree of tobacco with AOX knockout was more serious^27^.

As the center enzyme of complex IV in the electron transport chain, COX is related to the mitochondrial respiratory metabolism and ATP synthesis^39^. In the present study, the results showed that Al stress raised membrane lipid peroxidation and up-regulated the expression of *AhAOX* and *AhCOX*, which was not enough to inhibit PCD occurrence. The expression of *AhAOX* and *AhCOX* presented periodic fluctuation within 24 hours, speculating that they might be related to the biological clock. However, NO can enhance significantly the expression of *AhAOX* and *AhCOX*, protect the peanut root tip from ROS damage. As for how NO regulates their expression via a biological clock, it needs further study.

## Conclusions

**Figure 8:**
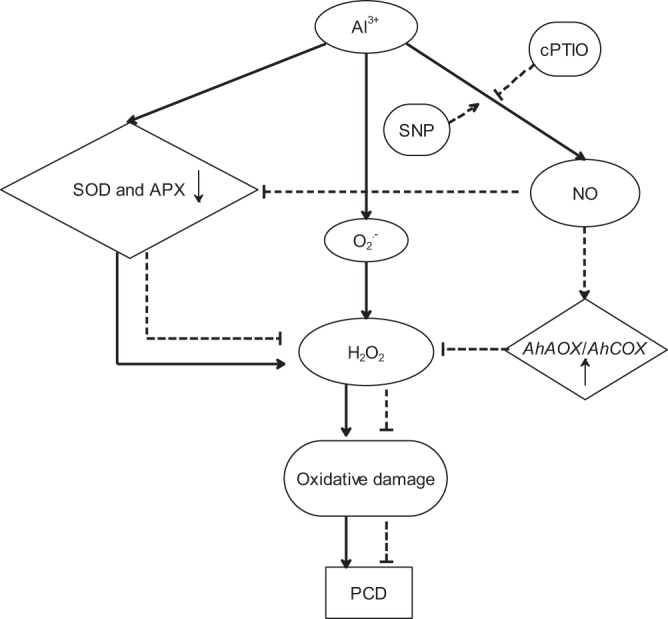
A proposed model for the role of NO in Al-induced PCD in the root tips of peanut. Al stress decreased the activities of SOD and APX, then led to the accumulation of ROS (O_2_^.−^ and H_2_O_2_) contents and oxidative damage, which resulted in PCD occurrence. NO partially prevented Al-induced decay of activities of SOD and APX, enhanced the expression of *AhAOX* and *AhCOX*, then reduced ROS production, which inhibited the production of PCD. The solid line represents Al^3+^ effect. The dashed line represents the inhibitory effect of NO on cell death. The sharp head represents promotion, whereas the flat head represents suppression.

Our results indicate that Al-induced PCD in the root tips of peanut is related to excessive ROS-induced mitochondrial physiological alterations by (Fig. 8). Al stress induces a large amount of mitochondrial ROS production in the root tips of peanut, causes mitochondrial membrane lipid peroxidation and mitochondria dysfunction, ultimately resulting in PCD production. But NO can enhance the expression of *AhAOX* and *AhCOX*, improve the activities of mitochondrial antioxidant enzymes (SOD, APX) to scavenge excess ROS, reduces the level of mitochondrial membrane lipid peroxidation to maintain the normal physiological function of mitochondria, thus inhibiting the occurrence of PCD.

## Methods

### Plant material and treatments

75% alcohol soaked in the seeds of Zhonghua 2 (Al-sensitive peanut variety) for 30 s. Peanut seeds were germinated for 4 days in the moist sand at 26 °C under dark condition. After the seed coat was removed, the germinated peanut seed was transplanted into a modified 1/5 Hoagland nutrient solution, which was changed every two days. The peanut seedlings of two leaf stage were pretreated for 24 h in a solution containing 0.1 mmol·L^−1^ CaCl_2_ (pH 4.2). Some seedlings were respectively treated with 100 μmol·L^−1^ AlCl_3_ containing 0.1 mmol·L^−1^ CaCl_2_ (pH 4.2) at different times (0 h, 1 h, 4 h, 8 h, 12 h, and 24 h). In addition, the following combination treatments were cultured for 12 h: ①0.1 mmol·L^−1^ CaCl_2_ (pH 4.2) (CK); ②100 μmol·L^−1^ AlCl_3_ (Al); ③100 μmol·L^−1^ AlCl_3_ + 200 μmol·L^−1^ SNP (Al + SNP); ④100 μmol·L^−1^ AlCl_3_ + 50 μmol·L^−1^ cPTIO (Al + cPTIO), cPTIO (2-(Carboxyphenyl)-4,4,5,5-tetramethylimidazoline-4-oxyl-3-oxide). After each treatment, 15 peanut root tips were collected.

### Determination of root cell death

Fresh roots were stained with 0.25% (w/v) Evans blue solution for 15 min. After washing with deionized water for 10 min, ten root tips (10 mm) were excised and digested or 1 h in 4 ml N,N-dimethylformamide at room temperature. The absorbance of Evans blue was measured at 600 nm^40^.

### Isolation of mitochondria from the root tips of peanut

According to Panda’s method^37^, mitochondria were separated from peanut root tips. After rinsing with distilled water, 5 ml mitochondrial extract buffer (0.3 mo1·L^−1^ mannitol, 25 mmol·L^−1^ MOPS-KOH (pH 7.8), 10 mmol·L^−1^ tricine, 8 mmol·L^−1^ cysteine, 1 mmol·L^−1^ EGTA, 0.1% (w/v) BSA, and 1% (w/v) PVP-40) were used to homogenized about 3 g fresh treated root tips on ice-bath. After 15 min centrifugation at 1 500 × g, the homogenate supernatant was centrifuged at 14 000 × g for 15 min. The precipitate was washed for 3 times by using mitochondrial suspension buffer (0.4 mo1·L^−1^ mannitol, 1 mmol·L^−1^ EGTA, 10 mmol·L^−1^ tricine, pH 7.2). The final pellet was resuspended with mitochondrial suspension buffer of appropriate volume. To detect the viability of mitochondria, a suspension stained with 0.02% Janus Green B was observed under oil microscope. According to the methods of Braidot *et al*.^41^ and Zhang^42^, the membrane potential and Cyt c value were detected, respectively. The method of Bradford^43^ was used to determine protein concentration, which represented the mitochondrial concentration.

### Assay of mitochondrial enzyme activities

The activity of mitochondrial SOD was determined by nitroblue tetrazolium (NBT) display method^44^. The total volume of 3 mL mixture included 1.5 mL of 0.05 mol·L^−1^ phosphate buffer (pH 7.8), 0.3 mL of 130 mmol·L^−1^ Met, 0.3 mL of 750 μmol·L^−1^ NBT, 0.3 mL of 100 μmol·L^−1^ EDTA-Na_2_, 0.3 mL of 20 μmol·L^−1^ riboflavin, 0.05 mL mitochondria extract, and 0.25 mL distilled water, respectively. 4 to 6 tubers were used as control, which the enzyme solution was replaced with buffer. After blending, two control tubes were placed in the dark, and other tubes under 4000 Lx fluorescent lamp were reacted for 20 min (the consistent light situation was required, the reaction time shortened at high temperature but the reaction time extension at low temperature). After the reaction ended, taking the control tube treated with dark as blank, OD_560_ of other tube were determined respectively. 50% suppression of NBT photoredox reaction was one enzyme activity unit. SOD activity = 2 × [OD_560_ (control) − OD_560_ (sample tube)] × volume of sample tube (mL)/OD_560_ (control) × Weight × volume of solution determined (mL). The unit of enzyme activity was U·μg^−1^ Pro. The activity of mitochondrial APX was determined as follows^45^. 3 mL reaction mixture contained 50 mmol·L^−1^ K_2_HPO_4_-KH_2_PO_4_ buffer (pH 7.0), 0.1 mmol·L^−1^ EDTA-Na_2_, 0.3 mmol·L^−1^ AsA, and 0.1 mL mitochondrial extract. After adding H_2_O_2_, the absorbance changes within 10 to 30 s at 290 nm were immediately determined at 20 °C and the reduction of AsA and enzyme activity in unit time were calculated. The unit of enzyme activity was μmol ASA·mg^−1^ Pro·min^−1^.

### Detection of O_2_^.−^ production rate and H_2_O_2_ content

The production rate of superoxide anion free radical (O_2_^.−^) was determined according to the method of Zhan *et al*.^15^. 0.5 mL of mitochondrial extract was put into a test tube, respectively. Then 0.5 mL of 50 mmol·L^−1^ phosphate buffer (pH 7.8) and 1 mL of 1 mol·L^−1^ hydroxylamine hydrochloride were added successively. After mixing, the mixture was kept for 1 h at room temperature. After 1 mL 17 mmol·L^−1^ aminobenzene sulfonic acid and 1 mL 7 mmol·L^−1^ alpha naphthylamine were added, mixing and displaying for 20 min at room temperature. With 0.5 mL phosphate buffer as a blank control, the absorbance at 530 nm was determined. According to the equation of hydroxylamine and O_2_^.−^, the formula [O_2_^.−^] = 2 [NO_2_^.−^] was used to express the production rate of O_2_^.−^ by stoichiometry. Nitrite content was calculated by the standard curve. According to the method of Sergiev^46^, H_2_O_2_ content was measured. 1 mL of mitochondrial extract, 2 mL of 1 mol·L^−1^ KI, and 1 mL of 0.1 mol·L^−1^ phosphate buffer (pH 7.0) were added to the test tube. According to a H_2_O_2_ standard curve, the absorbance at 390 nm measured after 20 min shake was converted into H_2_O_2_ concentrations.

### Determination of mitochondrial MDA content

0.2 mL mitochondrial extract (distilled water as a control) was put into a test tube and was added by 1 mL 0.6% thiobarbituric acid (TBA). Then it was bathed in boiling water for 15 min. After cooling, the homogenate was centrifuged at 1500 × g for 10 min to measure the absorbance at 532 nm, 600 nm, and 450 nm, respectively. Malondialdehyde (MDA) content was calculated according to the method of Zhan *et al*.^15^.

### Transcript analysis of *AhAOX* and *AhCOX* by quantitative RT-PCR

Peanut root tips were used to extract total RNA. Primer sequences of *AhAOX* (accession No. AES98635.1) were 5′-AGGTCACTCCGCAGGTTTCAG-3′ (forward) and 5′-AACTCCCTGGACAACAAGAACAAG-3′ (reverse). The primer sequences of *AhCOX* (accession No. AES58587.1) were 5′- TAGAGATCGGAGGTATTTGGCCC -3′ (forward) and 5′- CGCGAGTATAGCATGATGAGCC -3′ (reverse). After the reverse transcription of total RNA, the reaction system was as follows: 10 µL of 2 × SybrGreen qPCR Master Mix, 2 µL of cDNA, 1 µL of primer F (10 µmol·L^−1^), 1 µL of primer R (10 µmol·L^−1^), and 6 µL of ddH_2_O. The amplification procedure was conducted using at 95 °C for 10 min (initial), 95 °C for 10 s (melting), 55 °C for 10 s (annealing), and 72 °C for 20 s (extend) for 40 cycles. All quantifications were normalized to amplification of *Ahactin* (EU982407). The amount of gene expression was calculated by 2^−△△Ct^ relative quantitative analysis. The forward and reverse primer sequences of *Ahactin* were 5′-ATGGAGAAGATCTGGCATCATACC-3′ and 5′- TGGCAACATACATAGCAGGGG-3′, respectively.

### Hierarchical cluster analysis

Hierarchical cluster analysis was performed by Cluster 3.0 software^47^. Java Treeview was used to display the resulting tree figures^48^.

### Statistical analysis

All experiments were repeated for three times. The results were processed using one-way analysis of variance (ANOVA). The data represent the mean ± SD. The significant differences among the treatments were statistically evaluated by Student’s paired *t* test.
